# Magpies as Hosts for West Nile Virus, Southern France

**DOI:** 10.3201/eid1401.070630

**Published:** 2008-01

**Authors:** Elsa Jourdain, Michel Gauthier-Clerc, Philippe Sabatier, Océane Grège, Timothy Greenland, Agnès Leblond, Murielle Lafaye, Hervé G. Zeller

**Affiliations:** * Centre de Recherche de la Tour du Valat, Arles, France; † Institut Pasteur, Lyon, France; ‡ Ecole Nationale Vétérinaire de Lyon, Marcy l’Etoile, France; § Kalmar University, Kalmar, Sweden; ¶ Université Claude Bernard, Lyon, France; # French Space Agency, Toulouse, France

**Keywords:** West Nile virus, Pica pica, common magpie, serosurvey, viral shedding, sentinel bird, dispatch

## Abstract

European magpies (*Pica pica*) from southern France were tested for antibodies to West Nile virus (WNV) and viral shedding in feces during spring–autumn 2005. Results suggest that this peridomestic species may be a suitable sentinel species and a relevant target for additional investigations on WNV ecology in Europe.

West Nile virus (WNV, Flaviviridae, *Flavivirus*) is an arbovirus that principally infects a wide range of bird species, but spillover infections may occur in mammals, including horses and humans. In southern France, WNV was first reported during the 1960s in the Camargue, a wetland area with many types of birds. This virus was recently detected in the same area. It was responsible for 76 equine cases in 2000 and 32 equine cases in 2004. On the basis of ornithologic and epidemiologic data, several bird species were suggested as candidates for WNV amplification and emergence in the Camargue (*1*). Among these species, corvids may be of particular interest because several species of the family Corvidae have experimentally been shown to be highly competent for WNV transmission (*2*,*3*).

We studied the European magpie or common magpie (*Pica pica*) because this species is territorial and abundant in both wet and dry areas. Pilot serologic investigations conducted in the 2000 and 2004 Camargue outbreaks in horses suggested a high WNV seroprevalence in magpies (*4*,*5*). Furthermore, WNV was isolated in 2004 from a yearling magpie near a farm with clinical equine cases (*5*). The aim of our study was to better assess WNV seroprevalence in magpies in the Camargue area and detect WNV circulation during the postepizootic year of 2005.

## The Study

The study was conducted from late spring to early autumn 2005. Multicatch magpie traps, i.e., circular traps that catch <4 birds simultaneously, were set 1 day per week from July to September in different places within 3 areas (Figure, top panel). Area A contained dry and wet habitats in which some WNV equine cases were reported in 2004. Area B was a wetland in which most clinical equine cases occurred in 2004, and a WNV-positive magpie was detected in October 2004. Area C was a wetland in which a WNV-positive house sparrow (*Passer domesticus*) was detected in October 2004 (*5*). Additionally, some magpies were obtained in July and August 2005 from a crow ladder trap permanently set in area D, a dry area in which some horses had WNV infection in 2004. A few magpie nestlings were also sampled from their nest in May, June, and July 2005.

**Figure:**
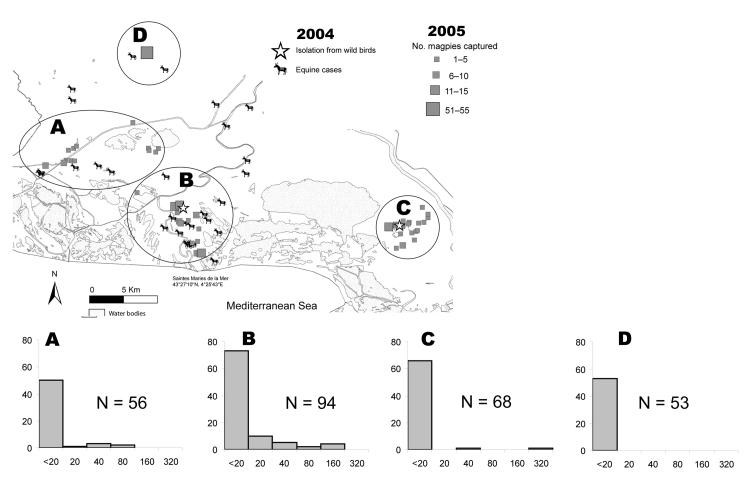
Area of Camargue, France, showing locations of magpie capture sites: site A (n = 56, with 24 adults, 32 juveniles, and 0 nestlings), site B (n = 94, with 34 adults, 57 juveniles, and 3 nestlings), site C (n = 68, with 17 adults, 34 juveniles, and 17 nestlings), and site D (n = 53, with 1 adult, 52 juveniles, and 0 nestlings). Confirmed equine and avian cases infected with West Nile virus in 2004 are also indicated. Histograms correspond to site designations and indicate serologic titers (x-axes) measured by using a microneutralization test plotted against no. birds (y-axes).

Flying birds were classified as juveniles or adults by using plumage criteria (*6*). All magpies were ringed, sampled (blood and cloacal swab), and released. Serum samples were first screened for immunoglobulin (Ig) G to WNV by using an indirect ELISA with horseradish peroxidase–conjugated anti-wild bird IgG (A140–110P; Bethyl Laboratories, Montgomery, TX, USA). Positive and doubtful samples were further tested by microneutralization by using the France 05.21/00 equine WNV strain (GenBank accession no. AY268132) and staining with crystal violet (*5*). Because the recapture rate of wild birds is usually low and WNV is excreted in feces of infected birds over a short period (*2*), we also tested for WNV RNA in feces of all 29 seropositive birds (i.e., 35 samples because some birds were captured several times) and 4 seronegative birds. Nucleic acid was extracted from cloacal swabs by using the QIAamp viral RNA mini kit (QIAGEN S.A., Courtaboeuf, France) and amplified with WNV-specific primers (*7*).

Of 271 magpies captured, 29 had WNV neutralizing antibodies at a titer >20, which confirmed a relatively high WNV seroprevalence (10.7%, 95% binomial confidence interval 7.3%–15.0%) in the Camargue magpie population. No seroconversion, i.e., a 4-fold increase in measured antibody titer, was detected in 46 (17%) magpies recaptured during the field season. Most titers were <80 (Figure, bottom panels), and WNV-positive birds recaptured in the summer (n = 5) showed titers stable over time. These findings, and the fact that adults (26/76, 34.3%) were more frequently seropositive than juveniles (3/173, 1.7%) (p<0.001, by χ^2^ test), suggest past exposure to the virus.

Because antibodies to WNV are believed to remain detectable in birds for >1 year (*8*,*9*), adult magpies that were seropositive in this study had probably been exposed to WNV within the past few years either during the recent 2004 epizootic circulation or before this time. The first possibility is supported by the fact that WNV-positive birds were particularly abundant at site B, in which most clinical equine cases were detected in 2004. Because maternal transmission of antibodies to WNV was reported in birds (*10*,*11*), detection of 3 juvenile magpies with low antibody titers may also be explained by the 2004 WNV circulation. However, a cloacal sample from a seropositive juvenile magpie captured in early August 2005 was positive by nested PCR, which suggests that WNV was circulating among wild birds in 2005. Unfortunately, infectious WNV could not be isolated from this sample.

## Conclusions

This serosurvey confirmed that a relatively high proportion of the Camargue magpie population has been exposed to WNV. Because magpies are sedentary, with only limited dispersal, seropositive birds in this study had likely been exposed to WNV in the Camargue area. Although serologic data suggested past exposure to the virus, detection of WNV-specific RNA by nested PCR in a cloacal swab suggests that WNV was circulating in the Camargue in 2005. No other evidence of WNV transmission was obtained in this serosurvey or by the national surveillance network of captive sentinel ducks or chickens, and no clinical equine cases were reported.

Our results suggest that magpies might be sensitive indicators of WNV enzootic activity in the Camargue. Although trapping magpies may be challenging because these birds are extremely wary and quickly learn how to avoid traps, surveillance of juvenile birds might be useful. Permanently set crow ladder traps with captive sentinel magpies to attract wild birds might be the most efficient way to capture large numbers of juvenile birds. This method would enable detection of seroconversion in captive magpies, and analysis by reverse transcription–PCR of cloacal swabs from free-ranging birds could be used as a supplemental WNV surveillance tool.

Further investigations are needed to better understand whether magpies are frequently exposed to WNV or whether observed seroprevalence is merely the result of a long history of virus circulation in the Camargue. Blood meal analyses of likely mosquito vector species, e.g., *Culex pipiens* L. and *Cx*. *modestus* Ficalbi (*12*,*13*), may help answer this question, although nonvectorial transmission may occur in this scavenger bird species (*14*). Other serologic surveys in the Camargue suggest that magpies have higher prevalence levels of WNV than other sedentary species (*4**,**5*; E. Jourdain, unpub. data). However, because only a few selected species were targeted and sample size was small for most of them, a survey of a wider range of bird species is needed to enable better comparisons. Other corvid species, such as the carrion crow (*Corvus corone*) or the jackdaw (*C*. *monedula*), would be relevant targets.

WNV has also been reported in magpies in Russia (*15*), which suggests that these birds might be useful for WNV surveillance in other European transmission foci. However, because WNV epidemiology is complex and highly variable between sites, local epidemiologic studies should be performed before magpies are used as sentinel birds in other areas.
